# Contemporary Strategies and Outcomes of Dedicated Chronic Total Occlusion Percutaneous Coronary Intervention Programs: A Prospective Multicentre Registry

**DOI:** 10.1155/2021/8042633

**Published:** 2021-12-07

**Authors:** Maarten Vanhaverbeke, Ward Eertmans, Wouter Holvoet, Ief Hendrickx, Keir McCutcheon, Christophe Dubois, Joseph Dens, Johan Bennett

**Affiliations:** ^1^Department of Cardiovascular Medicine, University Hospitals Leuven, Leuven, Belgium; ^2^Department of Cardiology, Ziekenhuis Oost-Limburg, Genk, Belgium; ^3^Department of Cardiovascular Sciences, KU Leuven, Leuven, Belgium

## Abstract

**Background:**

The field of CTO PCI is expanding, but successful and safe percutaneous coronary intervention (PCI) of coronary chronic total occlusions (CTO) requires a substantial resource and experience investment. We aimed to assess temporal trends in strategies and outcomes of 2 dedicated programs for CTO PCI.

**Methods:**

Between 2011 and 2020, 920 CTO PCI procedures were prospectively included at 2 referral centres in Belgium. Temporal trends were assessed, and logistic regression models were built to identify predictors of outcome.

**Results:**

Despite an increase in lesion complexity (the J-CTO score increased from 1.3 in year 1 to 1.7–2.0 in years 8-9, *p* < 0.001), technical success improved from 70.0% to 85.6% in year 9 (*p* value for trend <0.001). We observed the most significant improvement starting at years 3-4 (OR 2.3 in year 4 versus year 1, *p*=0.018). Together with an increase in success rates and lesions complexity, there was an increase in the use of dual injections, retrograde approaches, the number of balloons and stents, and the use of microcatheters. Conversely, there was a decrease in large bore access, an increase in radial approach, and a shift towards contemporary dissection/reentry techniques. This strategy resulted in a stable major complication rate of 4.7% (*p* value for trend 0.33). The rate of coronary procedure-related myocardial injury was high (71.0%) and was associated with the use of more intracoronary devices.

**Conclusions:**

Three to four years after initiation of a dedicated CTO PCI program with 50 CTO PCIs per year, consistent high technical success and low complication rates are achieved using contemporary strategies.

## 1. Introduction

Chronic total occlusions (CTO) of the coronary arteries are identified in 15–25% of patients with coronary artery disease undergoing coronary angiography [1]. Percutaneous coronary intervention (PCI) of CTOs has shown to reduce angina and ischemia burden in prospective clinical studies [2, 3]. CTO PCI currently has a class IIa (level of evidence B) indication in patients with stable angina despite optimal medical therapy and in patients with a large area of ischemia [4]. The impact of CTO PCI on hard clinical endpoints is still under investigation [5].

The introduction of new techniques to approach CTO PCI, such as the hybrid algorithm, have improved success rates over the years [6, 7]. Therefore, CTO PCI has been increasingly performed when indicated. Two Belgian centres initiated a dedicated CTO PCI program during which patients were prospectively enrolled. The aim of the current study was to assess contemporary trends in the strategies, approach, and outcomes of CTO PCI in Belgium, including technical success and safety. The results from this study can guide other hospitals with or without on-site cardiac surgery to establish and optimize a dedicated CTO PCI program.

## 2. Methods

A dedicated CTO program was set up in two tertiary Belgian centres: Ziekenhuis Oost-Limburg (ZOL) Genk and the University Hospitals Leuven. Patients diagnosed with a CTO undergoing an attempt at percutaneous revascularization in both centres were prospectively enrolled from September 2011 and December 2013 onwards, respectively. One dedicated CTO operator performed the procedures in each centre. The operators were trained interventional cardiologists with 3–15 years of experience in PCI. The CTO programs were initiated after technical training through workshops, followed by on-site proctoring (generally around 6 months) and further collaboration with local operators. This prospective registry was approved by the local ethical committee of both hospitals, and all patients provided informed consent. The hybrid algorithm for CTO PCI was applied whenever possible [6]. Reattempt procedures and additional CTO lesions in other coronary territories were considered as separate procedures.

Baseline patient and procedural characteristics were recorded. The Multicentre CTO Registry of Japan (J-CTO) score was assessed by the CTO operator [8]. Multivessel disease was defined as at least 2 coronary artery territories with a 50% stenosis or more. The successful technique for crossing the CTO was divided into 4 categories: antegrade wire escalation (AWE), antegrade dissection reentry (ADR), retrograde wire escalation (RWE), and retrograde dissection reentry (RDR). AWE included antegrade true lumen wiring and the parallel wire technique. ADR included subintimal tracking and reentry (STAR), limited antegrade subintimal tracking (LAST), and the use of the CrossBoss and/or Stingray system (Boston Scientific, Marlborough, MA). RDR included controlled antegrade and retrograde tracking and dissection (CART) and reverse CART.

Technical success was defined as TIMI-3 flow post-PCI with less than 30% residual stenosis. Procedural success was defined as technical success without major complications. Major adverse cardiovascular events (MACE) were defined as in-hospital death, stroke, or periprocedural MI. Major complications were defined as MACE plus perforation requiring treatment, major bleeding, or major vascular complications. Major vascular complications were defined as retroperitoneal hematoma, acute limb ischemia, and vascular bleeding, requiring prolonged hospitalization or transfusion. Coronary complications include coronary perforation, dissection, or periprocedural MI caused by angiographically visible thrombus.

Coronary procedure-related myocardial injury and periprocedural myocardial infarction (MI) were defined according to the fourth universal definition of myocardial infarction [9]. Periprocedural type 4a MI was defined as an increase of high-sensitivity troponin T of at least 5 times the upper reference limit (URL) (13 ng/L) or a 20% rise and >5x URL if already increased pre-PCI, in combination with symptoms of ischemia or ischemic ECG changes. Coronary procedure-related myocardial injury was defined as an increase of high-sensitivity troponin T values above the 99th percentile (13 ng/L) or a 20% rise if already increased pre-PCI. High-sensitivity troponin T levels were systematically determined pre-PCI and the day after PCI.

**Figure 1:**
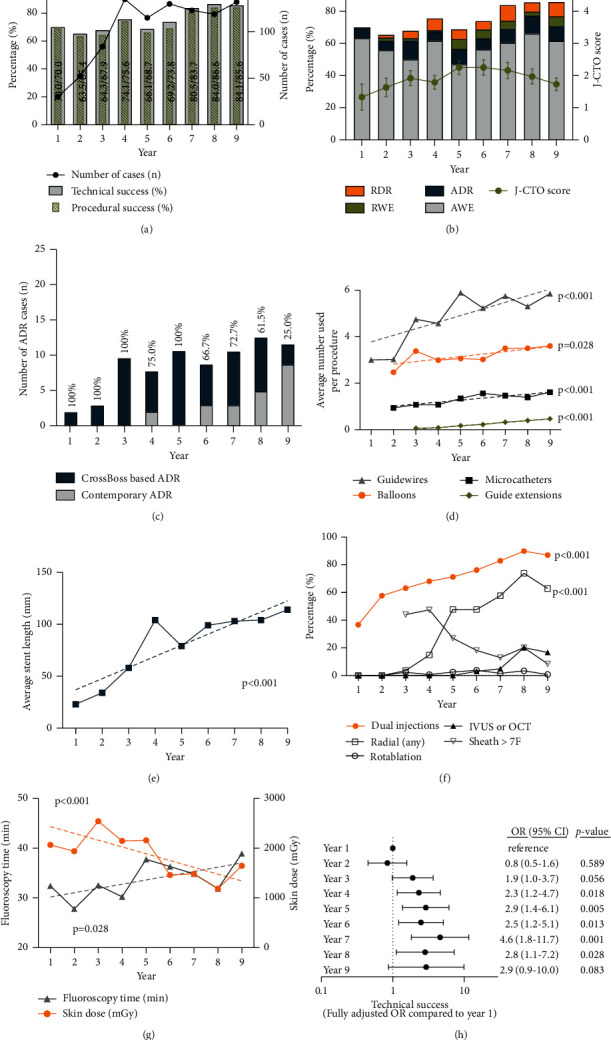
Temporal trends in the success rates and approaches of CTO PCI. (a) Significant improvement in technical success up to 85.6% and procedural success up to 84.1% in year 9 (*p* value for trend both <0.001). (b) Significant increase in the lesion complexity (increase in J-CTO score) (*p* value for trend <0.001), with an increase in retrograde and dissection/reentry techniques. (c) There was evolution from the use of the CrossBoss catheter to contemporary ADR techniques using dual or triple lumen microcatheters. (d) Temporal increase in the number of guidewires, microcatheters, and guide extensions (all *p* value for trend <0.001) and balloons (*p*=0.028). (e) Increase in the average stent length (*p* value for trend <0.001). (f) More comprehensive approaches with an increase in the use of dual injections and intracoronary imaging but contemporary with an increase in at least one radial access and a decrease in sheaths larger than 7F (all *p* for trend <0.001). (g) Temporal increase in fluoroscopy time (*p*=0.028) but decrease in the skin dose (*p* < 0.001). (h) Regression model adjusted for age, LV EF, vessel, J-CTO score, the use of microcatheters, dual injections, and intracoronary imaging showing a significant increase in technical success starting from year 3 and year 4, compared to year 1 as reference. ADR, antegrade dissection/reentry; AWE, antegrade wire escalation; IVUS, intravascular ultrasound; J-CTO, Multicentre CTO Registry of Japan score; LV EF, left ventricular ejection fraction; OCT, optical coherence tomography; OR, odds ratio; RDR, retrograde dissection/reentry; RWE, retrograde wire escalation.

**Figure 2:**
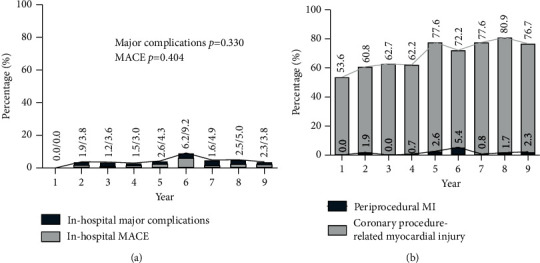
Temporal trends in the complications. (a) Stable rates of in-hospital major complications and MACE (cardiovascular death, stroke, and periprocedural MI) (*p* value for trend 0.330 and 0.404, respectively). (b) A significant temporal increase in the rate of coronary procedure-related myocardial injury (*p* value for trend <0.001), without an increase in periprocedural MI (*p* value for trend 0.310). MACE, major adverse cardiovascular events; MI, myocardial infarction; URL, upper reference limit.

Patient and lesion characteristics as well as complications in the first four years of the program versus thereafter were compared using Student's *t*-test or chi-square test (Tables 1 and 2). Formal significance testing for trends over time was performed by linear contrast analysis for continuous variables and the Cochran–Armitage test for dichotomous outcomes (Figures 1 and 2). Multivariable binary logistic regression models were built to identify independent predictors of technical success and complications, based on prespecified clinical variables. In the regression model, the year of enrollment was adjusted for the start of the CTO PCI program (2011 is year 1 for site 1 and 2013 is year 1 for site 2). All statistical analyses were performed in SPSS Statistics 24 (IBM, New York, USA).

Trends over time were statistically evaluated using linear contrast analysis for continuous variables and the Cochran–Armitage test for dichotomous outcomes. Details of the statistical analyses are available in the Supplementary Materials.

## 3. Results

From September 30, 2011, to April 1, 2020, 920 CTO PCI procedures were performed in 871 unique lesions (Table 1). The majority of patients were male (85.2%) and presented with stable angina (62.4%). The CTO was located in the RCA in the majority of patients (56.1%), and 33.4% of patients had a J-CTO score of 3 or higher. The CTO lesion characteristics are shown in Supplementary Table 1.

Success rates and procedural details are shown in Figure 1. Technical success improved significantly over time, from 70.0% in year 1 to 85.6% in year 9 (*p* value for trend <0.001, Figure 1(a)). Procedural success was equally high: an increase from 70.0% to 84.1% (*p* value for trend <0.001). Patient success increased from 77.8% to 84.0% (*p* value for trend 0.008).

Technical success improved despite a significant increase in lesion complexity over time. The J-CTO score increased from 1.3 ± 1.1 to 2.0 ± 1.3 in year 8 and 1.7 ± 1.1 in year 9 (*p* value for trend 0.004, Figure 1(b)). This increase was associated with a change in PCI strategy, with an increased use of dissection/reentry techniques and retrograde approaches. Retrograde approaches almost doubled from 8.6% in years 1–4 up to 15.5% in years 5–9 (*p* value for trend 0.048). Over time, CrossBoss catheters for an ADR approach were partially replaced by contemporary ADR techniques with use of Stingray or dual/triple-lumen microcatheters (Figure 1(c)).

Together with an increasing lesion complexity, a substantial increase over time was observed in the material used (Figure 1(d)), an increase in the average number of guidewires, microcatheters, guide extensions, and balloons. Likewise, average stent length increased significantly with 11 mm per year (Figure 1(e)).

Over time, procedures were more comprehensive with an increase in dual injections (87.0% in year 9, Figure 1(f)) and intracoronary imaging (up to 16.7% in year 9, *p* value for trend <0.001). The use of rotablation was low and stable at 2% (*p* value for trend 0.481). Despite the comprehensive approach, the procedures were less invasive with smaller sheath size (a decrease of sizes >7F, *p* < 0.001) and an increase in at least one radial access. With increasing lesion complexity, there was an increase in fluoroscopy time, but a decrease of the skin dose (Figure 1(g)).

The average rate of major in-hospital complications was 4.7% and for MACE 2.5% (Table 2). Despite increasing lesion complexity and more complex techniques, there was no significant increase in these outcomes over time (Figure 2(a)). The rate of coronary complications was 4.0%: 2.9% for the CTO vessel and 1.1% in the donor vessel (3.1% for retrograde cases). Three in-hospital deaths occurred: two patients died who presented with cardiogenic shock at the time of CTO PCI and the third patient died after coronary artery bypass grafting.

We observed a significant temporal increase in the rate of coronary procedure-related myocardial injury (*p* value for trend <0.001, Figure 2(b)), although there was no temporal increase in the rate of periprocedural MI (average 2.0%, *p* value for trend 0.310). The number of patients with periprocedural MI according to the fourth universal definition of myocardial infarction (with a cutoff of 5 times the upper reference limit, URL) was 18, compared to 11 patients according to the Academic Research Consortium-2 (ARC-2) definition (with a cutoff of 35 times the URL) [9].

We then identified predictors of outcome. Age, CTO length, the J-CTO score, the use of microcatheters, intracoronary imaging, and the year of enrollment, adjusted for the year of initiation of the CTO program, were significant univariate predictors of technical success (Supplementary Table 2). In a multivariable logistic regression model, age (OR 0.98 per year*p*=0.039), LV EF (OR 0.98 per %*p*=0.032), the J-CTO score (OR 0.43 per point increase*p* < 0.001), and the year of enrollment (OR 1.25 per year*p* < 0.001) were significant independent predictors of technical success (Supplementary Table 3). In this fully adjusted model, success rates improved significantly starting at year 3 (OR 1.9, 1.0–3.7, *p*=0.056) and year 4 (OR 2.3, 1.2–4.7, *p*=0.018) after initiation of the CTO program (Figure 1(h)). The J-CTO score was the only independent predictor of in-hospital major complications (OR 1.32 per point increase, *p*=0.032). In univariable analyses, the use of more intracoronary devices (microcatheters, intracoronary imaging, and guide extensions), lesion complexity, and the presence of preexisting ischemia were related to a higher rate of coronary procedure-related myocardial injury.

Finally, in univariate analysis, the site of enrollment was not related to the technical success rates (*p*=0.471). In multivariable analysis, adjusted for the year of enrollment and J-CTO score, there was no interaction between the year of enrollment and site of enrollment to predict technical success (*p*=0.196); both centres improved their success rates during the subsequent years of the registry.

## 4. Discussion

The accumulating evidence of CTO PCI to reduce angina and ischemia burden has stimulated the more widespread initiation of CTO PCI programs in regional hospitals and referral centres. We here sought to describe the contemporary trends in the approach and outcome after initiation of a dedicated CTO PCI program. Although lesion complexity increased over time, technical success improved significantly up to 85.6%. For the first time, we show that the largest improvement is observed 3-4 years after initiation of the CTO PCI program. This improvement coincided with the use of more materials (e.g., guidewires, microcatheters, balloons, and longer stents) and more comprehensive techniques over the years (dual injections, ADR, and retrograde approaches). On the other hand, the approach was contemporary with the use of smaller bore vascular access, radial access if possible, AWE with soft tip wires as first approach, and contemporary ADR techniques.

In general, our findings are consistent with other large-scale CTO registries [10–13]. Data from the European Registry of Chronic Total Occlusion (ERCTO) also show an increase in lesion complexity over time, together with an increase in material use and increasing procedural success (up to 87.6% in ERCTO versus 84.1% in the current study) [13]. Although an increase in the use of the CrossBoss crossing catheter is observed in the ERCTO registry, we observed a slight decrease of its use in favor of more contemporary ADR techniques, with dual and triple lumen microcatheters combined with the Stingray catheter. We here could also show that the largest improvement in success rates is observed 3-4 years after initiation of the CTO program. Within each centre, one experienced operator performed or lead all the procedures. With a yearly average of 50–60 patients per centre, a dedicated CTO program is to be expected running optimally after 3 years or approximately 150 procedures.

The improvement in technical success was not associated with an increase in complications in the current study. The major in-hospital complication rate of 4.7% in our series was similar to that in the ERCTO registry (4.4–5.2%), but lower than that reported in the OPEN-CTO registry (7%). In-hospital mortality was very low (0.3% compared to 0.9% in the OPEN-CTO registry) without any CTO PCI-related urgent surgical procedures (compared to 0.7%) [14]. Although lesion complexity in our series was similar to other studies (J-CTO score around 2.0), the contemporary approach with smaller caliber radial approach and soft tip wires may have led to a relatively lower complication rate.

Finally, periprocedural high-sensitivity troponin T levels were systematically collected in our prospective registry. The rate of periprocedural MI was similar to that observed in the OPEN-CTO registry (2.0% versus 2.6%). However, a high proportion of patients had coronary procedure-related myocardial injury (71.0%), defined by the fourth universal definition of myocardial infarction. The relevance of these findings remains unclear [15]. As also identified in other studies, a more aggressive approach and more complex lesions were related to a higher risk of coronary procedure-related myocardial injury. In addition, we now also show that preexisting ischemia is related to the presence of procedure-related myocardial injury, but not to periprocedural MI.

Our study has several limitations. First, this registry is moderate in size and patients were recruited in 2 centres only. The ongoing Belgian Working Group on CTO (BWGCTO) registry will continue to collect prospective data on contemporary CTO strategies and includes a larger number of participating centres [16]. Second, it is likely that success rates increased, despite increasing lesion complexity, because of the increased operator experience and improvement in tools and techniques. However, this remains only a hypothesis given the observational nature of this analysis. Moreover, the J-CTO score is determined by the operator, which may result in selection bias. Finally, although we included the use of intracoronary imaging in the regression model, IVUS or OCT was predominantly used to optimize stent implantation rather than guide wire crossing and may therefore not be a good predictor of technical success.

In conclusion, successful and safe CTO PCI requires a substantial resource and experience investment. We show here for the first time that 3-4 years after initiation of a dedicated CTO PCI program of 50–60 procedures per centre and per year, consistent high technical success rates are achieved despite an increase in lesion complexity.

## Figures and Tables

**Table 1:** Baseline patient characteristics.

	All (*n* = 920)	Years 1–4 (*n* = 301)	Years 5–9 (*n* = 619)	*P* value
Demographics				
Age	66 ± 10	65 ± 11	66 ± 10	0.201
Male	784 (85.2)	259 (86.0)	525 (84.8)	0.692
BMI	28.5 ± 8.1	29.6 ± 12.7	28.0 ± 4.4	**0.045**

Risk factors				
Current smoker	202 (22.0)	66 (21.9)	136 (22.0)	1.0
Hypertension	634 (68.9)	188 (62.5)	446 (72.1)	**0.004**
Hypercholesterolemia	784 (85.2)	230 (76.4)	554 (89.5)	**<0.001**
Diabetes	251 (27.3)	79 (26.2)	172 (27.8)	0.637

Medical history				
Prior MI	338 (36.7)	92 (30.6)	246 (39.7)	**0.007**
Prior PCI	436 (47.4)	132 (43.9)	304 (49.1)	0.140
Prior CABG	130 (14.1)	37 (12.3)	93 (15.0)	0.313
Prior stroke	59 (6.4)	22 (7.3)	37 (6.0)	0.474
PAD	193 (21.0)	62 (20.7)	131 (21.2)	0.931
CKD	211 (23.3)	64 (22.1)	147 (23.9)	0.556
Multivessel disease	489 (56.4)	165 (54.8)	324 (57.2)	0.518

LV function				
LV EF	55 ± 12	57 ± 12	54 ± 12	**<0.001**
LV EF ≤ 35%	83 (9.2)	21 (7.0)	62 (10.4)	0.113

CTO presentation				
ACS	136 (14.8)	43 (14.3)	93 (15.1)	0.768
Stable angina	570 (62.4)	185 (61.5)	385 (62.9)	0.716
Atypical chest pain	21 (2.3)	5 (1.7)	16 (2.6)	0.483
Asymptomatic	135 (15.0)	61 (20.3)	74 (12.3)	**0.002**
Silent ischemia	275 (32.5)	90 (30.0)	185 (33.8)	0.283

Target vessel				
LAD	248 (27.0)	89 (29.6)	159 (25.7)	0.235
CX	150 (16.3)	49 (16.3)	101 (16.3)	1.0
RCA	516 (56.1)	160 (53.2)	356 (57.5)	0.229
LMCA	6 (0.7)	3 (1.0)	3 (0.5)	0.399

ACS, acute coronary syndrome; BMI, body mass index; CTO, chronic total occlusion; CX, circumflex coronary artery; CKD, chronic kidney disease; LAD, left anterior descending artery; LV, left ventricular; LV EF, left ventricular ejection fraction; LMCA, left main coronary artery; PAD, peripheral artery disease; RCA, right coronary artery. Dichotomous variables are reported as number (percentages). Continuous variables are reported as means with standard deviation. Significant *p* values below 0.05 are shown in bold.

**Table 2:** In-hospital complications.

	All (*n* = 920)	Years 1–4 (*n* = 301)	Years 5–9 (*n* = 619)	*P* value
Any complication	86 (9.3)	23 (7.6)	63 (10.2)	0.230
Major complication	43 (4.7)	9 (3.0)	34 (5.5)	0.098
Major adverse cardiovascular events	23 (2.5)	4 (1.3)	19 (3.1)	0.175
In-hospital death	3 (0.3)	1 (0.3)	2 (0.3)	1.0
Stroke	2 (0.2)	1 (0.3)	1 (0.2)	0.548
Periprocedural MI	18 (2.0)	2 (0.7)	16 (2.6)	0.072

Coronary complications	37 (4.0)	13 (4.3)	24 (3.9)	0.724
CTO vessel	27 (2.9)	10 (3.3)	17 (2.7)	0.678
Donor vessel	10 (1.1)	3 (1.0)	7 (1.1)	1.0
Coronary perforation	28 (3.0)	10 (3.3)	18 (2.9)	0.838
Ellis type 2 or more	18 (2.0)	8 (2.7)	10 (1.6)	0.314
Requiring intervention	12 (1.3)	3 (1.0)	9 (1.5)	0.760

Vascular complications	30 (3.3)	6 (2.0)	24 (3.9)	0.166
Retroperitoneal hematoma	3 (0.3)	1 (0.3)	2 (0.3)	1.0
Hematoma > 5 cm	12 (1.3)	5 (1.7)	7 (1.1)	0.542
Pseudoaneurysm	12 (1.3)	0 (0)	12 (1.9)	**0.011**
Acute limb ischemia	3 (0.3)	0 (0.5)	3 (0.5)	0.555

Major vascular complication	9 (1.0)	3 (1.0)	6 (1.0)	1.0

Others				
Pericardial effusion	16 (1.7)	6 (2.0)	10 (1.6)	0.789
Major bleeding	9 (1.0)	3 (1.0)	6 (1.0)	1.0
Contrast-induced nephropathy	16 (1.7)	4 (1.3)	12 (1.9)	0.601

Myocardial injury				
hsTroponin T, baseline (ng/L)	15 (9–30)	18 (10–40)	14 (9–25)	**0.003**
hsTroponin T, day 1 (ng/L)	43 (22–110)	33 (18–90)	50 (24–127)	**<0.001**
Coronary procedure-related myocardial injury	462 (71.0)	154 (61.1)	308 (77.2)	**<0.001**

CTO, chronic total occlusion; hsTroponin T, high-sensitivity troponin T; MI, myocardial infarction. Troponin values are median with interquartile range. Dichotomous variables are reported as number (percentages). Significant *p* values below 0.05 are shown in bold.

## Data Availability

The data used to support the findings of this study are available from the corresponding author upon request.

## Supplementary Materials

Supplementary Materials Graphical abstract: in the current prospective multicentre registry from 2011 to 2020, 920 CTO procedures were analyzed. Technical success rates go up after 3-4 years after initiation of a coronary CTO PCI program, reaching 85.6% in year 9. Comprehensive but contemporary approaches with dual injections are safe without increase in major complications (4.7%). CTO, chronic total occlusion. Supplementary tables: PCI indications and CTO lesion characteristics (Supplementary Table 1). Univariate predictors of outcome (Supplementary Table 2). Multivariable logistic regression identifying independent predictors of in-hospital outcome (Supplementary Table 3).
