# Supplementary material for: Contemporary Strategies and Outcomes of Dedicated Chronic Total Occlusion Percutaneous Coronary Intervention Programs: A Prospective Multicentre Registry
Source: J Interv Cardiol. 2021 Dec 7;2021:8042633. doi: 10.1155/2021/8042633 (PMC8670896; doi:10.1155/2021/8042633)
Supplement: Supplementary Materials — Graphical abstract: in the current prospective multicentre registry from 2011 to 2020, 920 CTO procedures were analyzed. Technical success rates go up after 3-4 years after initiation of a coronary CTO PCI program, reaching 85.6% in year 9. Comprehensive but contemporary approaches with dual injections are safe without increase in major complications (4.7%). CTO, chronic total occlusion. Supplementary tables: PCI indications and CTO lesion characteristics (Supplementary Table 1). Univariate predictors of outcome (Supplementary Table 2). Multivariable logistic regression identifying independent predictors of in-hospital outcome (Supplementary Table 3). [file 8042633.f1.zip › 8042633.f1/Suppl_Tables_rev1.docx]

Supplemental Table 1: PCI indications and CTO lesion characteristics

|  | All (n=920) | Year 1-4 (n=301) | Year 5-9 (n=619) | *p-value* |
| --- | --- | --- | --- | --- |
| PCI indications |  |  |  |  |
| Angina | 501 (54.5) | 190 (63.1) | 311 (50.2) | **<0.001** |
| Ischemia | 314 (34.1) | 93 (30.9) | 221 (35.7) | 0.159 |
| ACS | 73 (7.9) | 28 (9.3) | 45 (7.3) | 0.299 |
| Ventricular arrhythmia | 38 (4.1) | 14 (4.7) | 24 (3.9) | 0.598 |
| Dyspnea | 13 (1.4) | 0 (0.0) | 13 (2.1) | **0.007** |
|  |  |  |  |  |
| CTO lesion characteristics |  |  |  |  |
| CTO length (mm) | 22 ± 13 | 20 ± 7 | 23 ± 15 | **0.001** |
| J-CTO Score | 2.0 ± 1.3 | 1.8 ± 1.2 | 2.1 ± 1.3 | **<0.001** |
| Entry shape | 421 (45.8) | 109 (36.2) | 312 (50.5) | **<0.001** |
| Calcification | 465 (50.6) | 138 (45.8) | 327 (52.9) | 0.049 |
| Bending | 270 (29.4) | 68 (22.6) | 202 (32.7) | **0.002** |
| Occlusion length | 498 (54.2) | 166 (55.1) | 332 (53.7) | 0.724 |
| Retry | 158 (17.2) | 46 (15.3) | 112 (18.1) | 0.306 |
| J-CTO > 3 | 307 (33.4) | 79 (26.2) | 228 (36.9) | **0.001** |

CTO, chronic total occlusion. Dichotomous variables are reported as number (percentages). Continuous variables are reported as means with standard deviation.

Supplemental Table 2: Univariate predictors of outcome.

|  | **Technical success** | | **Major complications** | | **Acute myocardial injury** | | **Periprocedural MI** | |
| --- | --- | --- | --- | --- | --- | --- | --- | --- |
|  | *OR* | *P* | *OR* | *P* | *OR* | *P* | *OR* | *P* |
| **Patient characteristics** | | |  |  |  |  |  |  |
| Age | 0.98 (0.97-0.99) | ***0.007*** | 1.01 (0.98-1.03) | *0.505* | 1.01 (0.99-1.02) | *0.505* | 1.02 (0.98-1.07) | *0.325* |
| LV EF | 0.99 (0.97-1.00) | *0.078* | 0.99 (0.97-1.02) | *0.528* | 1.01 (0.99-1.02) | *0.330* | 0.98 (0.95-1.02) | *0.272* |
| CKD | 0.88 (0.61-1.26) | *0.491* | 1.14 (0.56-2.30) | *0.722* | 1.32 (0.88-1.98) | *0.175* | 1.27 (0.45-3.60) | *0.654* |
| Angina* | 0.99 (0.723-1.34) | *0.931* | 0.79 (0.43-1.46) | *0.449* | 1.16 (0.83-1.63) | *0.384* | 0.66 (0.26-1.70) | *0.392* |
| Ischemia* | 1.01 (0.73-1.40) | *0.953* | 0.93 (0.48-1.78) | *0.824* | 1.56 (1.08-2.24) | ***0.017*** | 0.55 (0.18-1.67) | *0.289* |
| **Lesion characteristics** | | |  |  |  |  |  |  |
| CTO: RCA | 0.86 (0.63-1.17) | *0.336* | 2.09 (1.06-4.12) | ***0.034*** | 1.21 (0.86-1.70) | *0.267* | 2.79 (0.91-8.54) | *0.072* |
| ISR | 1.20 (0.71-2.03) | *0.504* | 0.65 (0.20-2.13) | *0.474* | 0.85 (0.48-1.49) | *0.562* | 1.10 (0.25-4.86) | *0.901* |
| CTO length | 0.97 (0.96-0.98) | ***<0.001*** | 1.03 (1.09-1.04) | ***0.003*** | 1.01 (0.99-1.02) | *0.266* | 1.04 (1.02-1.06) | ***<0.001*** |
| J-CTO score | 0.46 (0.40-0.54) | ***<0.001*** | 1.37 (1.07-1.74) | ***0.012*** | 1.21 (1.06-1.39) | ***0.007*** | 1.40 (0.97-2.03) | *0.072* |
| **Approach** | | |  |  |  |  |  |  |
| Dual injections | 0.86 (0.60-1.23) | *0.407* | 1.48 (0.68-3.23) | *0.330* | 1.76 (1.21-2.57) | ***0.003*** | 2.69 (0.62-11.81) | *0.189* |
| Microcatheter | 0.47 (0.22-1.00) | ***0.049*** |  |  | 1.82 (1.03-3.24) | ***0.041*** |  |  |
| IVUS or OCT | 2.61 (1.10-6.17) | ***0.029*** | 0.69(0.18-3.16) | *0.688* | 3.02 (1.16-7.82) | ***0.023*** |  |  |
| Retrograde# | 0.72 (0.20-2.56) | *0.613* | 1.56 (0.57-4.23) | *0.386* | 1.39 (0.71-2.74) | *0.337* | 0.80 (0.10-6.46) | *0.834* |
| **Other variables** | | |  |  |  |  |  |  |
| Year of enrollment$ | 1.20 (1.12-1.29) | ***<0.001*** | 1.10 (0.96-1.26) | *0.161* | 1.09 (1.01-1.18) | ***0.030*** | 1.18 (0.96-1.44) | *0.116* |
| Technical success  Site of enrollment | 1.13 (0.82-1.56) | 0.471 | 0.36 (0.19-0.66)  0.65 (0.33-1.29) | ***0.001***  *0.220* | 1.02 (0.67-1.53)  2.58 (1.81-3.67) | *0.941*  ***<0.001*** | 0.18 (0.07-0.47)  0.42 (0.13-1.31) | ***<0.001***  *0.135* |
|  |  |  |  |  |  |  |  |  |

ACS, acute coronary syndrome; ISR, in-stent restenosis; IVUS, intravascular ultrasound; LVEF, left ventricular ejection fraction; OCT, optical coherence tomography. * CTO PCI Indication. # Successful crossing technique. $ Adjusted for initiation of the CTO program. Shown are odds ratios with 95% confidence interval. Empty parameters were not calculated because of low numbers or near-complete separation.

Supplemental Table 3: Multivariable logistic regression identifying independent predictors of in-hospital outcome

|  | **Technical success** | | **In-hospital major complications** | |
| --- | --- | --- | --- | --- |
|  | OR | *p*-value | OR | *p*-value |
|  |  |  |  |  |
| Age | 0.98 (0.96-1.00) | **0.039** | 1.01 (0.98-1.04) | 0.604 |
| LV EF (%) | 0.98 (0.97-1.00) | **0.032** | 0.99 (0.97-1.02) | 0.538 |
| CTO: RCA | 1.13 (0.78-1.64) | 0.515 | 1.93 (0.94-3.95) | 0.073 |
| J-CTO score | 0.43 (0.37-0.51) | **<0.001** | 1.32 (1.02-1.71) | **0.032** |
| Microcatheter | 0.51 (0.22-1.22) | 0.129 |  |  |
| Dual injections | 1.40 (0.88-2.21) | 0.156 | 0.88 (0.37-2.10) | 0.767 |
| IVUS or OCT | 1.78 (0.69-4.62) | 0.236 | 0.67 (0.16-2.94) | 0.599 |
| Year of enrollment* | 1.25 (1.14-1.37) | **<0.001** | 1.12 (0.97-1.30) | 0.119 |

CTO, chronic total occlusion; RCA, right coronary artery; IVUS, intravascular ultrasound; LV EF, left ventricular ejection fraction; OCT, optical coherence tomography. * Adjusted for initiation of the CTO program. Shown are odds ratios with 95% confidence interval.
