# Supplementary figures and images for: Contemporary Strategies and Outcomes of Dedicated Chronic Total Occlusion Percutaneous Coronary Intervention Programs: A Prospective Multicentre Registry
Source: J Interv Cardiol. 2021 Dec 7;2021:8042633. doi: 10.1155/2021/8042633 (PMC8670896; doi:10.1155/2021/8042633)

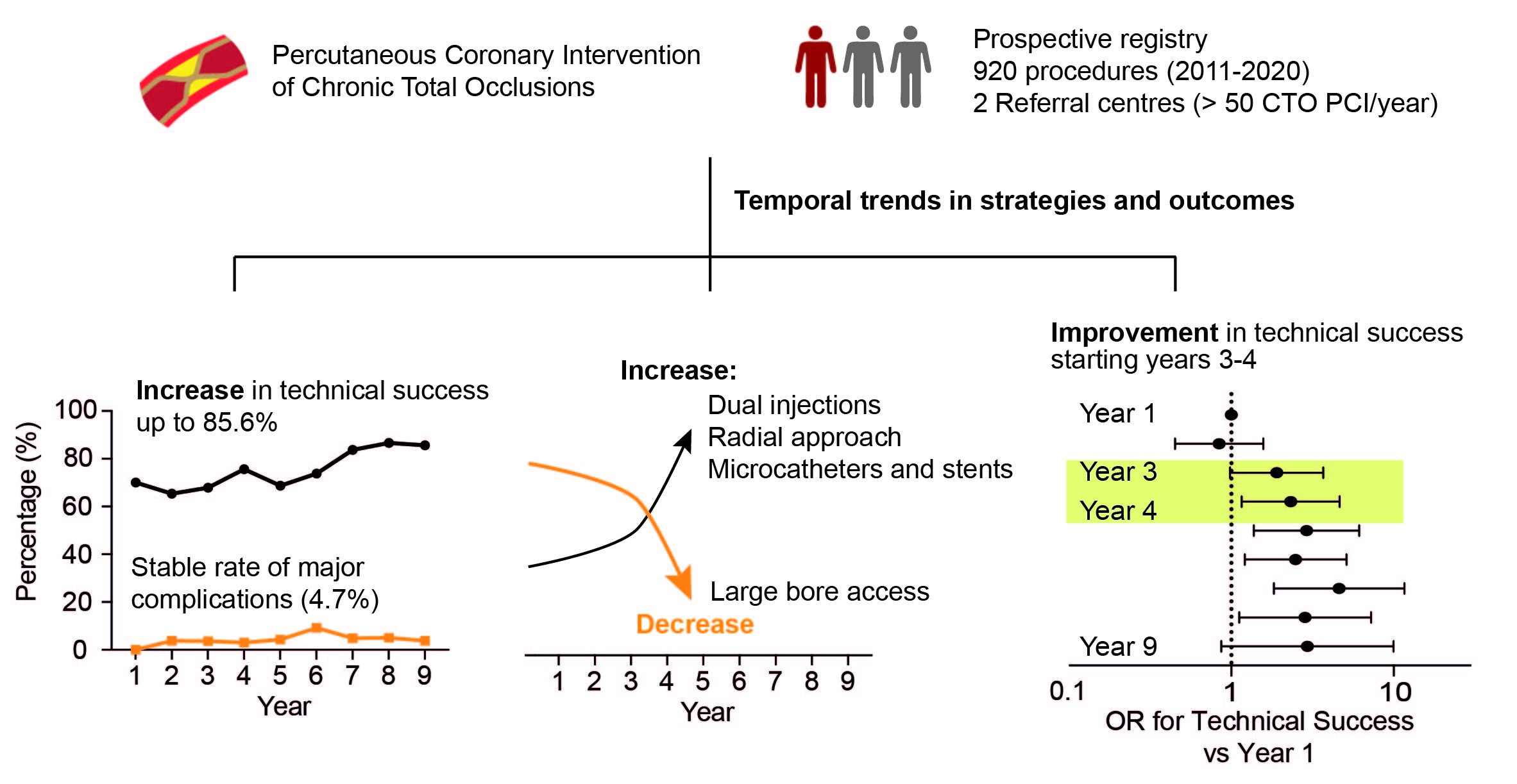

Supplement: Supplementary Materials — Graphical abstract: in the current prospective multicentre registry from 2011 to 2020, 920 CTO procedures were analyzed. Technical success rates go up after 3-4 years after initiation of a coronary CTO PCI program, reaching 85.6% in year 9. Comprehensive but contemporary approaches with dual injections are safe without increase in major complications (4.7%). CTO, chronic total occlusion. Supplementary tables: PCI indications and CTO lesion characteristics (Supplementary Table 1). Univariate predictors of outcome (Supplementary Table 2). Multivariable logistic regression identifying independent predictors of in-hospital outcome (Supplementary Table 3). [file 8042633.f1.zip › 8042633.f1/Graph_abs_CTO (2).jpg]
